# Supplementary material for: Large language models are less effective at clinical prediction tasks than locally trained machine learning models
Source: J Am Med Inform Assoc. 2025 Mar 8;32(5):811–22. doi: 10.1093/jamia/ocaf038 (PMC12012369; doi:10.1093/jamia/ocaf038)
Supplement: ocaf038_Supplementary_Data [file ocaf038_supplementary_data.pdf]

Supplementary Materials

Large Language Models are Less Effective at Clinical Prediction Tasks than Locally Trained Machine Learning Models

Authors

Katherine E. Brown, PhD[1], Chao Yan, PhD[1], Zhuohang Li, MS[2], Xinmeng Zhang, BS[2], Benjamin X. Collins, MD, MS[1], You Chen, PhD[1,2], Ellen Wright Clayton, MD, JD[3,4,5], Murat Kantarcioglu, PhD[6], Yevgeniy Vorobeychik, PhD[7], Bradley A. Malin, PhD[1,2,8]

Affiliations

[1]Department of Biomedical Informatics, Vanderbilt University Medical Center, Nashville, Tennessee
[2]Department of Computer Science, Vanderbilt University, Nashville, Tennessee
[3]Law School, Vanderbilt University, Nashville, Tennessee, USA
[4]Department of Health Policy, Vanderbilt University Medical Center, Nashville, Tennessee
[5]Department of Pediatrics, Vanderbilt University Medical Center, Nashville, Tennessee, USA
[6]Department of Computer Science, Virginia Tech, Blacksburg, Virginia
[7]Department of Computer Science, Washington University, St. Louis, Missouri
[8]Department of Biostatistics, Vanderbilt University Medical Center, Nashville, Tennessee

Corresponding Author

Katherine E. Brown, PhD
Department of Biomedical Informatics
Vanderbilt University Medical Center
Suite 1475, 2525 West End Avenue
Nashville, TN 37203, USA
Email: katherine.brown@vumc.org

S.1 Additional Dataset Information

This section details information about the evaluated datasets from BIDMC and VUMC. This includes the feature name, feature type, possible values (if categorical), and the unit and normal range provided to GPT.

Table S1. Detail for the features in each dataset. Italicized features are considered demographic variables.

Dataset	Feature Name	Type w/o generalization/suppression	Values	Unit & Normal Range provided to GPT
MIMIC	No. Days in the ED in the past <30/90/365> days	Integer		
	No. Days as an Inpatient in the Hospital in the past <30/90/365> days	Integer		
	No. Days in the ICU in the past <30/90/365> days	Integer		
	Age	Float		Years
	Gender	Categorical	Male/Female	
	Race	Categorical	White, Asian, Black/African-American, Hispanic/Latino, Native Hawaiian or Other Pacific Islander, Multiple Race/Ethnicity, Patient Declined to Answer, ?	
	Temperature at Triage	Continuous		degrees Fahrenheit (Normal: 97-99 degrees Fahrenheit)
	Heartrate at Triage	Continuous		beats per minute (Normal: 60-100 beats per minute)
	Respiratory Rate at Triage	Continuous		breaths per minute (Normal: 12-20 breaths per minute)
	Oxygen Saturation at Triage	Continuous		% (Normal: 97%-100%)
	Systolic Blood Pressure at Triage	Continuous		mm Hg (Normal: 90-120 mm Hg)
	Diastolic Blood Pressure at Triage	Continuous		mm Hg (Normal: 60-80 mm Hg)
	Reported Pain Level at Triage	Continuous		Reported Pain Level at Triage (Possible Values Range: 0 = no

				pain, 10+ = severe pain)
	Emergency Severity Index	Categorical	1 (most severe), 2, 3, 4, 5 (least severe)	(Possible Values Range: 1 = most severe, 5 = least severe)
VUMC	*Race*	Categorical	Non-Hispanic White, Non-Hispanic Black, Hispanic, Other Race/Ethnicity, Multi	
	Age	Continuous	Age of patient	Years
	Sex	Categorical	Male/Female	
	Day of Week	Categorical	Monday, Tuesday, Wednesday, Thursday, Friday, Saturday, Sunday	
	Insurance	Categorical	Type of insurance held (Private, self-pay, public)	
	Area Deprivation Index of Residence	Continuous	ADI of ZIP code listed as residence	
	Viewed Discharge Instructions	Continuous	Audit Log Information	Accesses
	Order Reconciliation Section accessed	Continuous		Accesses
	Obstetrics redirected print group viewed	Continuous		Accesses
	Patient device removed	Continuous		Accesses
	OR report printed GetSurgicalRecord webservice accessed	Continuous		Accesses
	Report with patient data viewed	Continuous		Accesses
	Communication sent	Continuous		Accesses
	Report with patient data printed	Continuous		Accesses
	Notes viewed	Continuous		Accesses
	Order sets accessed	Continuous		Accesses
	After Visit Summary Viewed	Continuous		Accesses
	Printing occurred	Continuous		Accesses
	Patient based RW report exported	Continuous		Accesses

Table S2. Demographic and class label distribution

		Full Dataset	LLM Evaluation
VUMC	Label (Positive/Negative)	9821/19,059	522/978
	Sex (Male/Female)	16,025/12,855	704/796
	Race (White/Not White)	21,973/6907	1140/360
	Age (18-49/50+)	13,386/15,49	659/841
MIMIC	Label (Positive/Negative)	23840/369736	92/1408
	Sex (Male/Female)	179,086/214,490	714/786
	Race (White/Not White)	231,291/162,285	875/625
	Age (18-49/50+)	168,133/225,443	640/860

S.2 LLM Prompt Information

This section details the specific prompt template used for both tasks. We provide the system message to help contextualize the LLM to the task and the question posed to the LLM. Input features are provided in JSON after the system message and before the question.

Table S3. LLM Prompt Template.

Dataset	Prompt Component	Prompt Text
VUMC	System Message	You are an expert at predicting patient discharge from a hospital given their demographic information and audit log statistics for the past 24 hours.
	Question	Question: Will the patient be discharged on the described day?<br>Provide your answer in the following format: {"answer": 0(no)/1(yes), "predicted_probability": (probability that answer is correct, in decimal, round to three decimal places)}<br>DO NOT produce any additional text.
MIMIC	System Message	You are an experienced attending physician who gives precise forecasting of emergency department patient outcomes based on patient presentation at triage.
	Question	Question: Will the patient be admitted to an ICU within 24 hours?<br>Provide your answer in the following format: {"answer": 0(no)/1(yes), "predicted_probability": (probability that answer is correct, in decimal, round to three decimal places)}<br>DO NOT produce any additional text.

S.3 Data Generalization Diagrams

This section details the hierarchical generalization diagrams for both datasets. Unless otherwise noted, the bottom row depicts the feature value without generalization or suppression. Ascending levels depict a higher level of generalization until the features are suppressed, denoted by *.

Figure S1. Hierarchical generalization diagram for VUMC dataset.

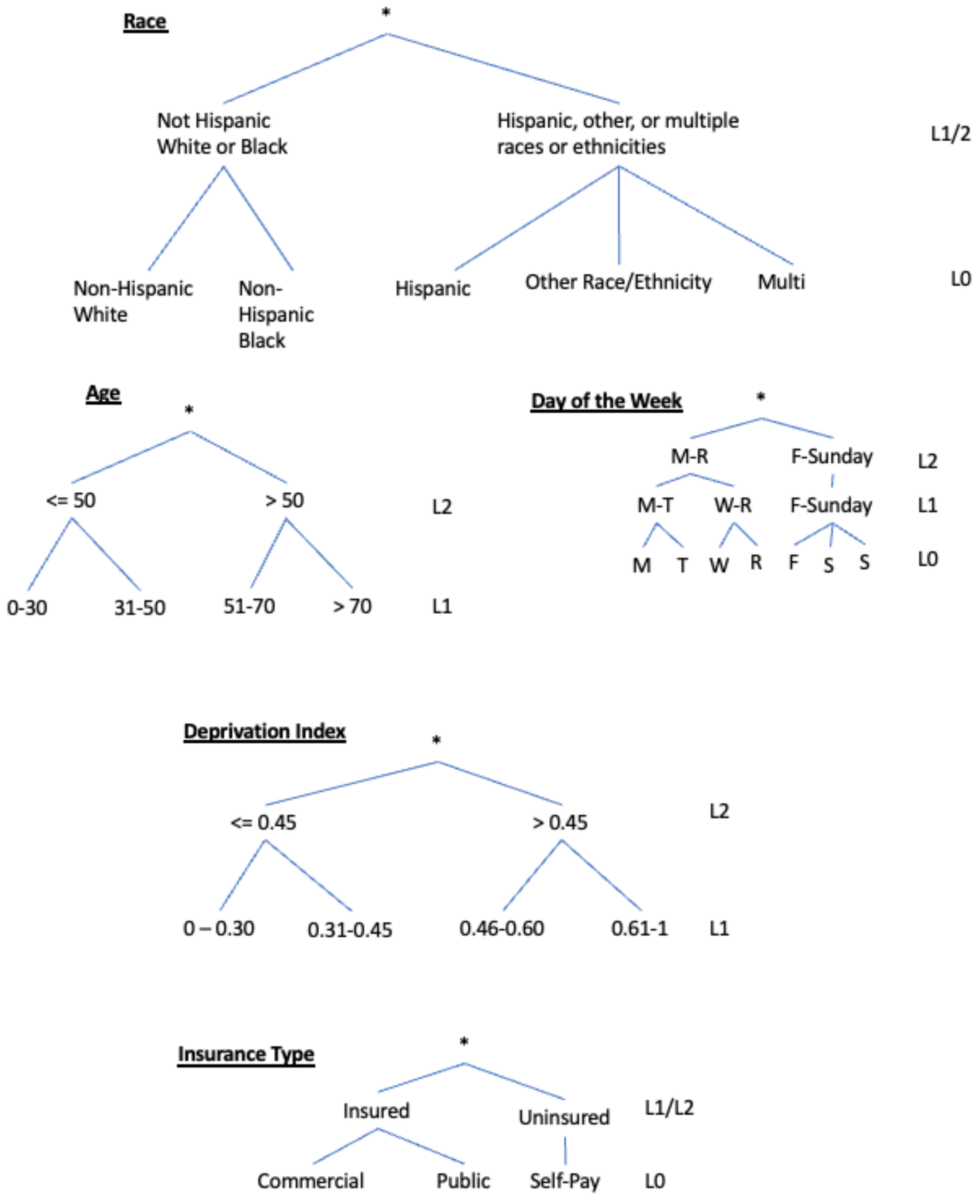

Figure S2. Hierarchical generalization diagram for MIMIC-IV dataset.

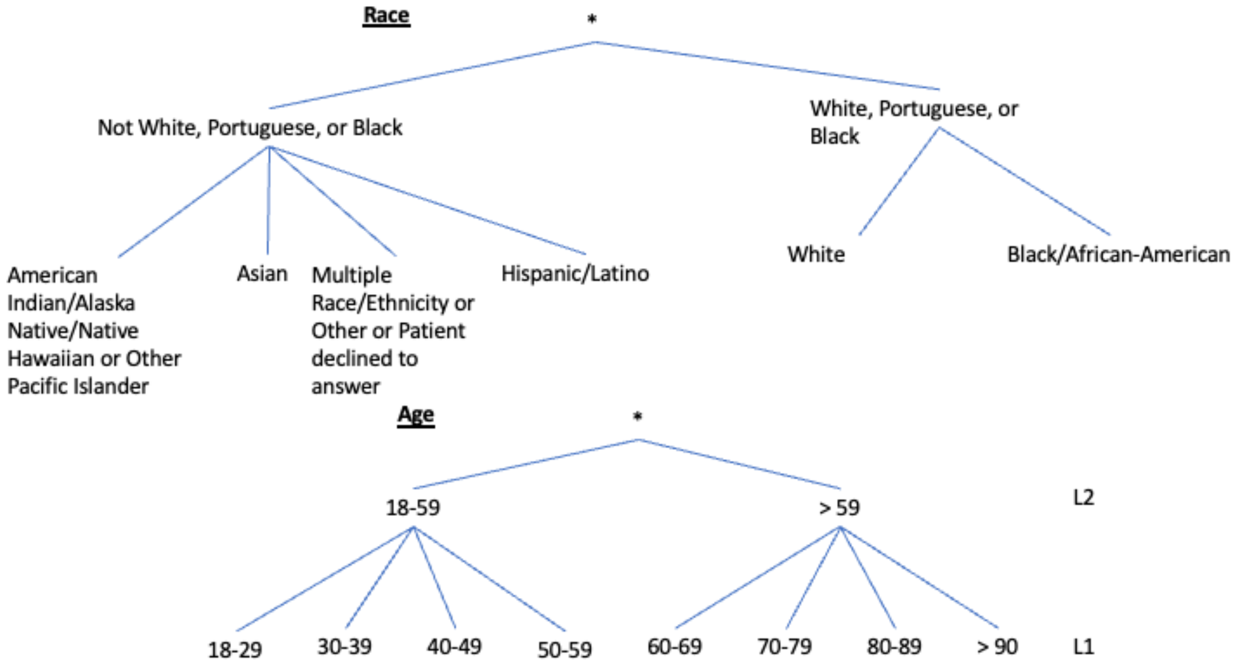

S.4 Additional Results

We now detail the sensitivity analysis of varying the number of few-shot examples provided to the LLM. Let *n* be the number of few-shot examples provided to the LLM. We evaluated values of *n* starting at *n* = 5 up to *n* = 25 in increments of 5 if that value of *n* did not cause the input to surpass the model's context length. For the VUMC data on GPT-3.5 (Figure S3, Panel A), *n = 5* was the optimal number of few-shot examples. For the MIMIC data on GPT-3.5 (Figure S3, Panel B), any few-shot examples with *n > 5* resulted in the input (including the *n* few-shot examples) surpassing the model's context length. For VUMC data on GPT-4 (Figure S3, Panel C) and MIMIC data on GPT-4 (Figure S3, Panel D), we note that no visually noticeable difference in performance occurred for n > 5. Thus, since there is no noticeable advantage to selecting more than 5 few-shot examples and to maintain consistency between model evaluations, we selected a total of 5 few-shot examples for our main results.

Figure S3. This figure depicts the ROC Curves when selecting up to 25 RAG-based few-shot examples. If a value of *n* caused the input to surpass the model's context length, this evaluation of *n*-shot examples is not included.

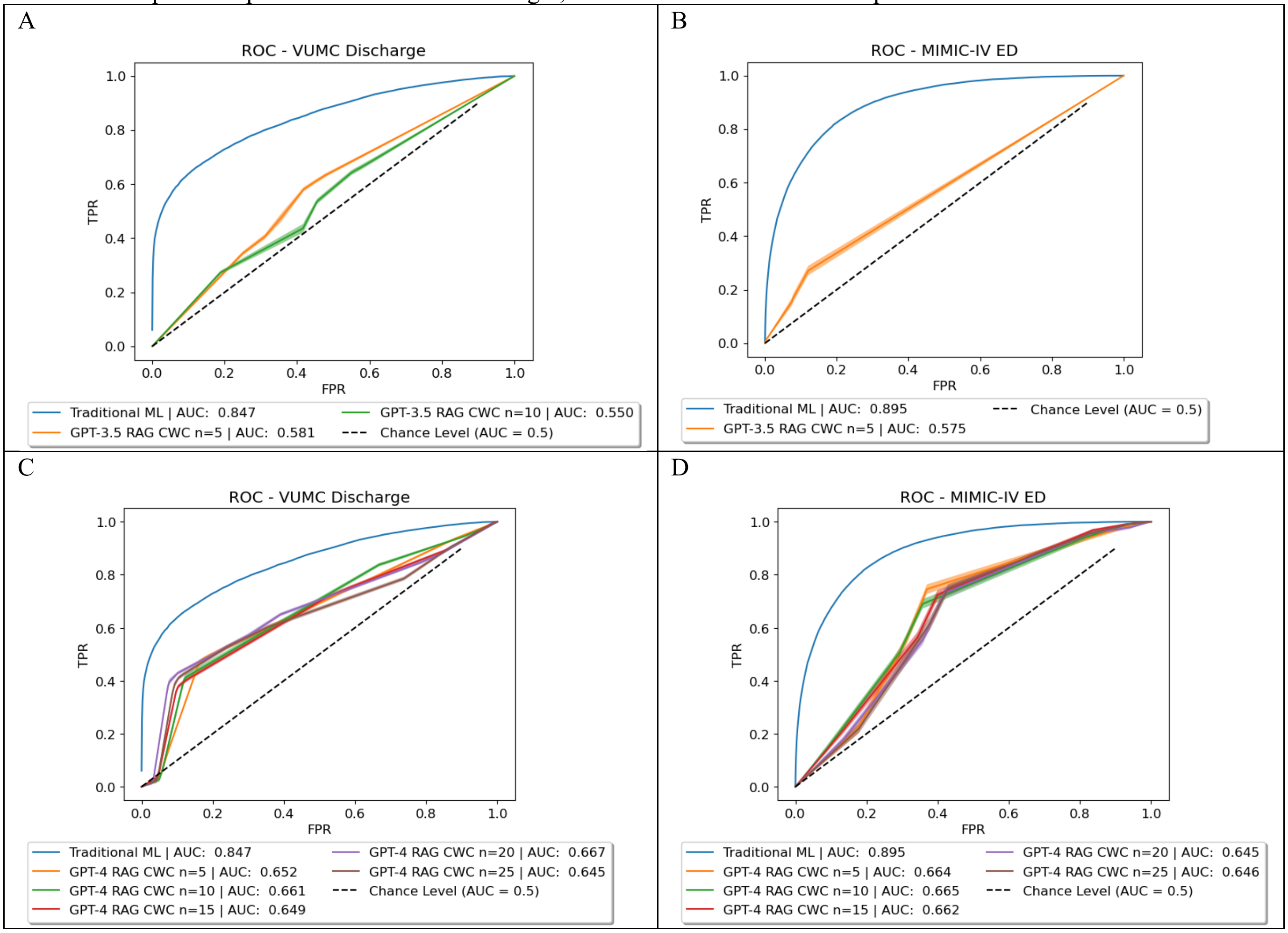

We now detail additional results from the fairness analysis. This includes heatmaps of the p-values from Welch's t-test to evaluate which model is fairer under average absolute odds difference or absolute value of statistical parity difference. We consider $p < 0.05$ as statistically significant.

Figure S4. This figure is for the VUMC data using (A) average absolute odds difference on zero-shot prompting, (B) average absolute odds difference on RAG-based few-shot prompting, (C) absolute value of statistical parity difference on zero-shot prompting, (B) absolute value of statistical parity difference on RAG-based few-shot prompting. Let $model_1$ be the model given by the row and $model_2$ be the model given by the column. Then,

$\mathcal{H}_0: \mu_{model_1} = \mu_{model_2}$ and $\mathcal{H}_1: \mu_{model_1} < \mu_{model_2}$. A lower p-value indicates $model_1$ is fairer (AAOD, |SPD|) than $model_2$.

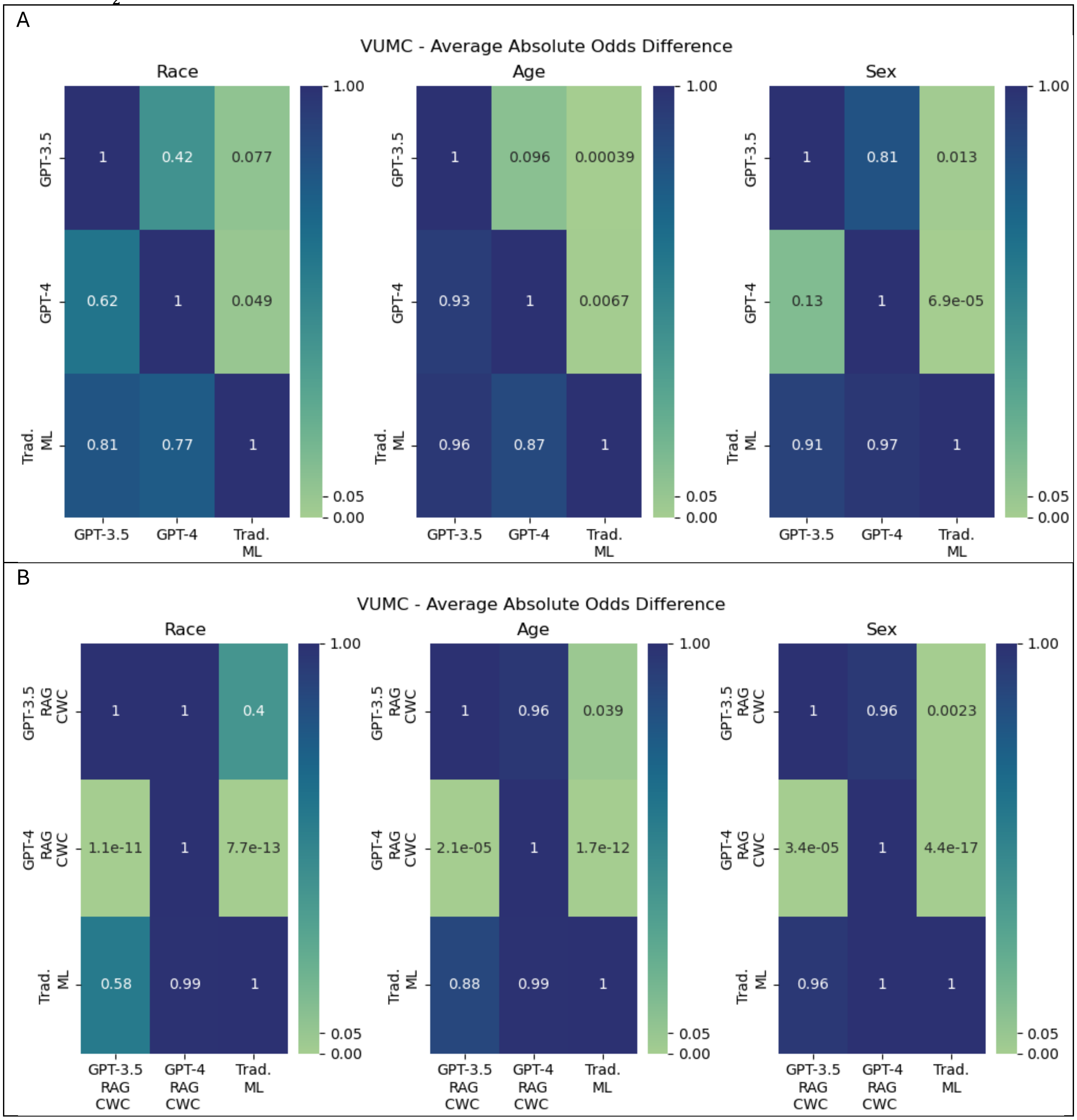

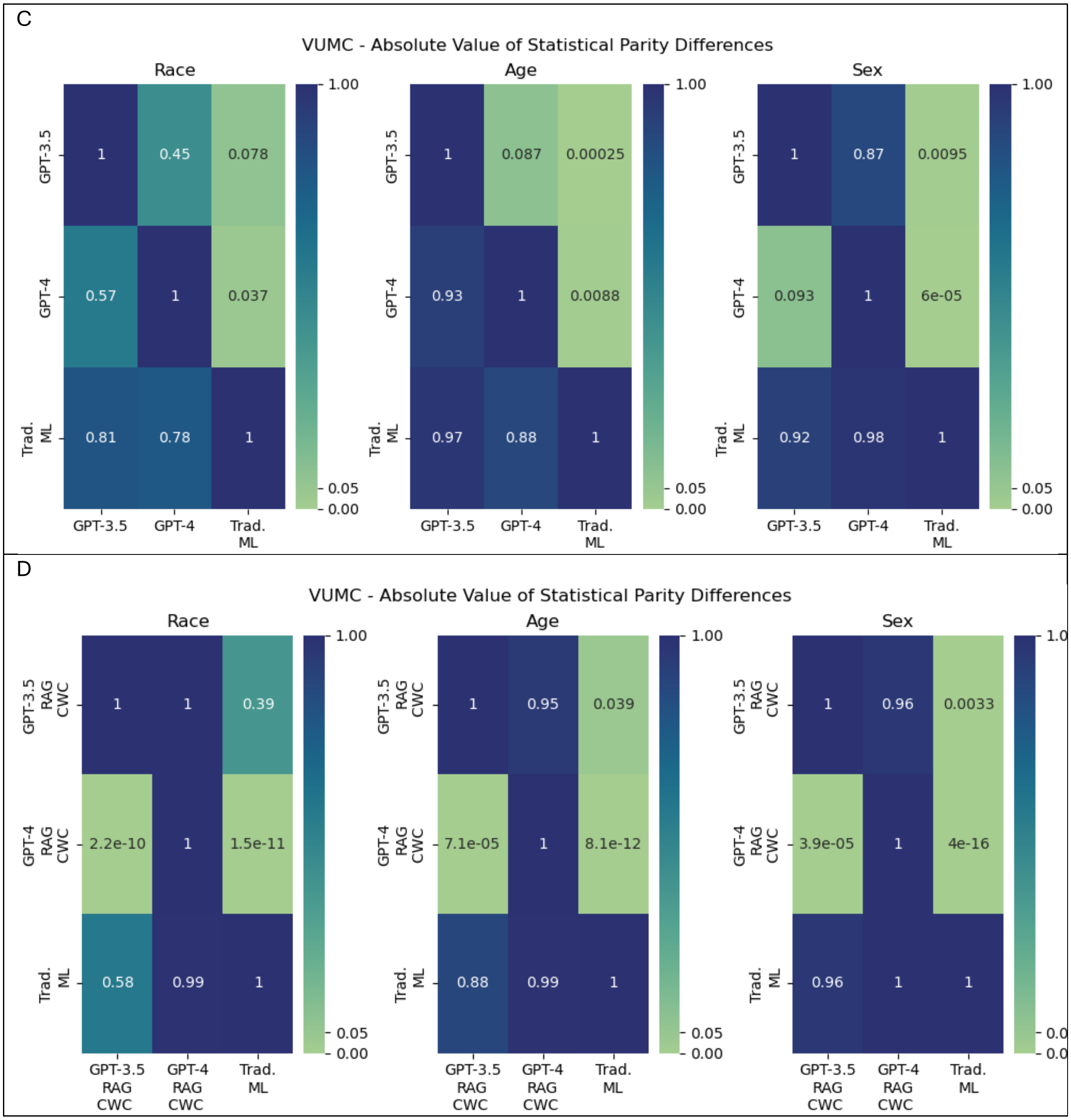
C
VUMC - Absolute Value of Statistical Parity Differences
Race
Age
Sex
GPT-3.5
GPT-4
Trad. ML
1
0.45
0.078
0.57
0.037
0.81
0.78
0.087
0.00025
0.93
0.0088
0.97
0.88
0.87
0.0095
0.093
6e-05
0.92
0.98
1.00
0.05
0.00
D
VUMC - Absolute Value of Statistical Parity Differences
GPT-3.5 RAG CWC
GPT-4 RAG CWC
0.39
2.2e-10
1.5e-11
0.58
0.99
0.95
0.039
7.1e-05
8.1e-12
0.96
0.0033
3.9e-05
4e-16

Figure 4. This figure is for the MIMIC-IV data using (A) average absolute odds difference on zero-shot prompting, (B) average absolute odds difference on RAG-based few-shot prompting, (C) absolute value of statistical parity difference on zero-shot prompting, (B) absolute value of statistical parity difference on RAG-based few-shot prompting. Let $model_1$ be the model given by the row and $model_2$ be the model given by the column. Then, $\mathcal{H}_0: \mu_{model_1} = \mu_{model_2}$ and $\mathcal{H}_1: \mu_{model_1} < \mu_{model_2}$. A lower p-value indicates $model_1$ is fairer (AAOD, |SPD|) than $model_2$.

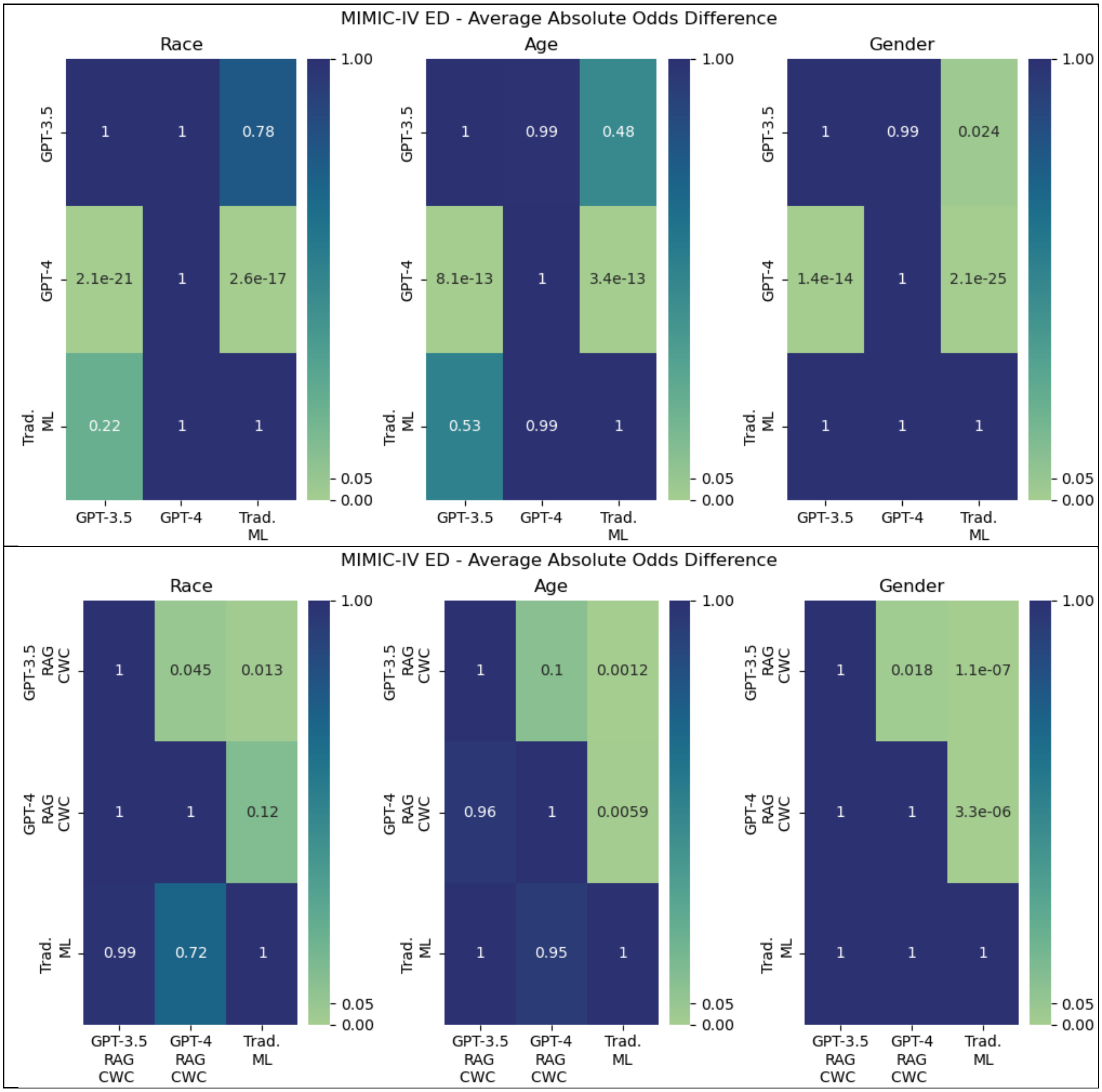

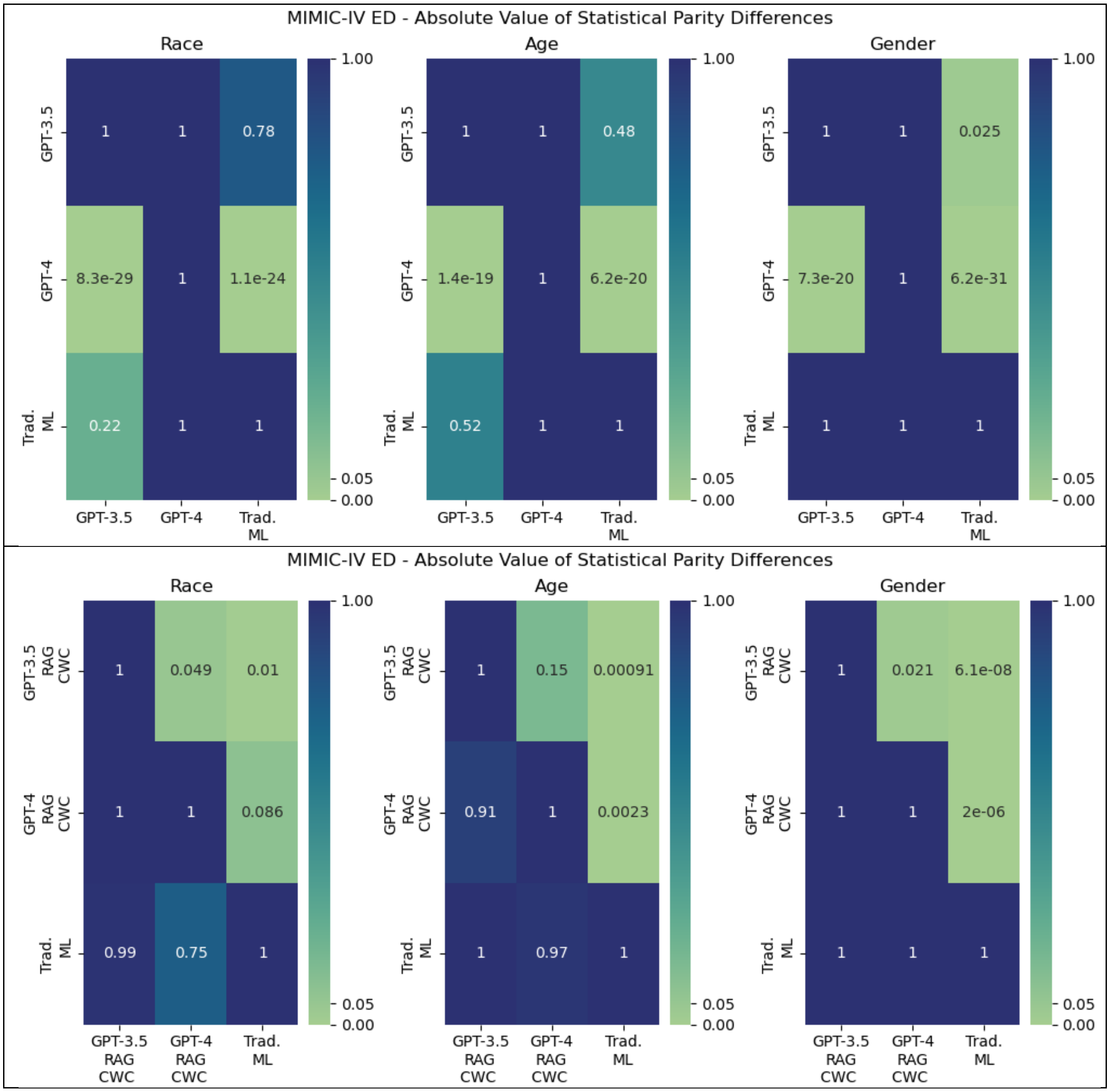
MIMIC-IV ED - Absolute Value of Statistical Parity Differences
Race
Age
Gender
GPT-3.5
GPT-4
Trad. ML
1
1
0.78
8.3e-29
1
1.1e-24
0.22
1
1
0.48
1.4e-19
6.2e-20
0.52
0.025
7.3e-20
6.2e-31
1.00
0.05
0.00
MIMIC-IV ED - Absolute Value of Statistical Parity Differences
GPT-3.5 RAG CWC
GPT-4 RAG CWC
0.049
0.01
0.086
0.99
0.75
0.15
0.00091
0.91
0.0023
0.97
0.021
6.1e-08
2e-06
